# Dehydroepiandrosterone Prevents H_2_O_2_-Induced BRL-3A Cell Oxidative Damage through Activation of PI3K/Akt Pathways rather than MAPK Pathways

**DOI:** 10.1155/2019/2985956

**Published:** 2019-04-28

**Authors:** Longlong Li, Yao Yao, Zhihao Jiang, Jinlong Zhao, Ji Cao, Haitian Ma

**Affiliations:** Key Laboratory of Animal Physiology and Biochemistry, College of Veterinary Medicine, Nanjing Agricultural University, Nanjing 210095, China

## Abstract

Dehydroepiandrosterone (DHEA) is a popular dietary supplement that has well-known benefits in animals and humans, but there is not enough information about the mechanisms underlying its effects. The present study aimed at investigating these mechanisms through *in vitro* experiments on the effects of DHEA on rat liver BRL-3A cells exposed to oxidative stress through H_2_O_2_. The findings showed that DHEA increased the antioxidant enzyme activity, decreased ROS generation, and inhibited apoptosis in H_2_O_2_-treated cells. These effects of DHEA were not observed when the cells were pretreated with known antagonists of sex hormones (Trilostane, Flutamide, or Fulvestrant). Furthermore, treatment with estradiol and testosterone did not have the same protective effects as DHEA. Thus, the beneficial effects of DHEA were associated with mechanisms that were independent of steroid hormone pathways. With regard to the mechanism underlying the antiapoptotic effect of DHEA, pretreatment with DHEA was found to induce a significant decrease in the protein expression of Bax and caspase-3 and a significant increase in the protein expression of PI3K and p-Akt in H_2_O_2_-treated BRL-3A cells. These effects of DHEA were abolished when the cells were pretreated with the PI3K inhibitor LY294002. No changes were observed on the p-ERK1/2, p-p38, and p-JNK protein levels in H_2_O_2_-induced BRL-3A cells pretreated with DHEA. In conclusion, our data demonstrate that DHEA protects BRL-3A cells against H_2_O_2_-induced oxidative stress and apoptosis through mechanisms that do not involve its biotransformation into steroid hormones or the activation of sex hormone receptors. Importantly, the protective effect of DHEA on BRL-3A cells was mainly associated with PI3K/Akt signaling pathways, rather than MAPK signaling pathways.

## 1. Introduction

Oxidative stress, which is caused by an increase in the production of reactive oxygen species (ROS), plays an important role in the development of liver diseases like fatty liver, alcohol liver, and liver injury [1]. Oxidative stress affects cell functioning by damaging lipids, proteins, and enzymes, and this subsequently leads to the apoptosis of the affected cells [2]. Hydrogen peroxide (H_2_O_2_), which is a prominent ROS, is closely involved in the induction of liver oxidative stress [3]. High H_2_O_2_ levels are responsible for lipid peroxidation and DNA damage, which eventually lead to the apoptosis of hepatic cells [4, 5]. The H_2_O_2_-induced apoptosis of hepatocytes involves the inhibition of antioxidative mechanisms and apoptosis-associated regulatory proteins like Bcl-2 family proteins and caspases. Thus, inhibition of proapoptotic pathways might be a feasible way of preventing or stalling liver damage caused by excess H_2_O_2_ production. Furthermore, since oxidative stress has been implicated in the majority of liver injuries [6, 7], another treatment strategy for liver injury might be the use of active antioxidant molecules that ameliorate liver oxidative stress.

The incidence of and susceptibility to liver diseases is known to increase with age [8]. There is some speculation that the aging-related degenerative changes observed in humans is associated with an aging-related marked decline in the levels of dehydroepiandrosterone (DHEA). In fact, the decrease in circulating DHEA levels is associated with multiple metabolic consequences including autoimmune diseases, aberrations in lipid metabolism, type 2 diabetes, and oxidative stress-related diseases [9]. Recently, DHEA was reported to exhibit antioxidative effects under conditions of acute as well as chronic oxidative stress [10–12], and these antioxidant effects have been confirmed through *in vivo* [13, 14] and *in vitro* [15] experiments, including our recent study in which DHEA treatment was found to protect various types of cells against oxidative damage [16, 17]. Although these beneficial effects of DHEA are known, there is not enough information about the mechanisms through which it exerts these effects.

DHEA is essentially a precursor protein that has the potential to transform into estrogens or androgens in organs such as the kidney, brain, gonads, and liver [18, 19], and it can exert various physiological effects by binding receptors, such as the estrogen receptor, androgen receptor, and other highly specific receptors, in the target tissues. Some studies have reported that DHEA executes its effects mainly through conversion into sex steroids and activation of androgen or estrogen receptors [20–22]. For example, our recent study found that DHEA reduced lipid droplet accumulation in primary hepatocytes from the chicken through its biotransformation into steroid hormones [23], and Mills et al. showed that DHEA promotes the healing of cutaneous injuries by activating estrogen receptors [20]. In contrast, there is also some evidence that the positive effects of DHEA are independent of the activation of sex steroid receptors [24–26]. This means that DHEA may exert its physiological effects as a neurosteroid by directly binding to neurotransmitter receptors. However, the mechanisms underlying its effects and its efficacy remain unclear in the absence of sufficient supporting data. The present study sought to fill in this gap in the literature.

Based on the findings of the literature so far, the aims of the present study were to determine whether DHEA protects H_2_O_2_-exposed BRL-3A cells from oxidative stress and apoptosis and to identify the signaling pathways and mechanisms that may be involved in the effects of DHEA. We believe that these findings will shed light on the antioxidative mechanisms of DHEA, which may have potential for the treatment of oxidative stress-induced conditions in humans.

## 2. Materials and Methods

### 2.1. Reagents

Testosterone, estradiol, DHEA, dimethyl sulfoxide (DMSO), and penicillin-streptomycin were obtained from Sigma-Aldrich (St. Louis, USA). The bicinchoninic acid (BCA), ROS, and cell apoptosis assay kits were from the Beyotime Institute of Biotechnology (Shanghai, China). The rabbit anti-caspase 3, anti-Bax, anti-3*β*-HSD (3*β*-hydroxysteroid dehydrogenase), and anti-17*β*-HSD (17*β*-hydroxysteroid dehydrogenase) antibodies were obtained from Abcam (London, England). The rabbit ERK1/2, anti-PI3K (phosphatidylinositol-3-kinase), anti-Akt, anti-JNK, anti-phosphorylated (p)-ERK1/2, anti-p-Akt, and anti-p-JNK antibodies were purchased from Cell Signaling Technology (CST, Boston, USA). The rabbit anti-tubulin *β*, anti-p38, and anti-p-p38 antibodies, as well as the goat anti-rabbit horseradish peroxidase-conjugated IgG were obtained from Boster Biological Technology Co. (Wuhan, China). Fulvestrant (an estrogen receptor (ER) antagonist), Flutamide (an androgen receptor (AR) antagonist), Trilostane (a 3*β*-HSD inhibitor), and LY294002 (PI3K inhibitor) were obtained from Selleck Chemicals (Houston, USA). Testosterone, estradiol, and DHEA were dissolved in DMSO, which was then diluted in DMEM-F12 medium. The DMSO content in the working solutions was less than 0.1% of the total volume.

### 2.2. Cell Culture

Rat liver BRL-3A cells (ATCC, Manassas, USA) were passaged every 3-4 days in DMEM-F12 medium (HyClone Laboratories Inc., Los Angeles, USA) containing 10% fetal bovine serum, 1% of 100 U/mL penicillin, and 100 *μ*g/mL streptomycin at 37°C in a 5% CO_2_ atmosphere. The cells were used for the experiments when they reached 80-90% confluence.

### 2.3. Testosterone and Estradiol Measurement by Radioimmunoassay

BRL-3A cells were grown in 6-well plates (1 × 10^6^ cells/well) and treated with DHEA at doses of 0, 1, 10, or 100 *μ*M DHEA for 24 h. The BRL-3A cells were then harvested and ultrasonically disrupted on ice. Next, the cells were centrifuged at 2500 ×g for 10 min at 4°C. The supernatants were extracted, and the estradiol and testosterone contents were measured using radioimmunoassay kits (Beifang Biotechnology Research Institute, Beijing, China).

### 2.4. ROS Analysis

BRL-3A cells were pretreated with vehicle, 10 *μ*M Trilostane, 10 *μ*M Flutamide, or 1 *μ*M Fulvestrant for 60 min. The cells were then treated with DHEA (0, 1, 10, or 100 *μ*M), testosterone (12.0 nM), or estradiol (6.3 nM) for 24 h and subsequently treated with 150 *μ*M H_2_O_2_ for another 2 h. Intracellular ROS content was analyzed with the fluoroprobe 2′,7′-dichlorodihydrofluorescein diacetate (H2DCF-DA) and dihydroethidium (DHE) as shown in our recent reports [16, 17]. Briefly, BRL-3A cells were incubated with H2DCF-DA for 30 min at 37°C, washed three times, and resuspended in cold PBS, after which they were immediately subjected to flow cytometry analysis with FACSCalibur™ (Becton Dickinson, San Jose, USA).

### 2.5. Cell Apoptosis Analysis

The cell treatments were the same as described in Section 2.4 for ROS analysis, and cell apoptosis was examined as reported in our studies [16, 17]. Briefly, BRL-3A cells were collected and washed with cold PBS, placed in 195 *μ*L Annexin V-FITC binding solution, incubated in 5 *μ*L Annexin V-FITC and 10 *μ*L propidium iodide in the dark for 30 min, and immediately subjected to flow cytometry analysis with FACSCalibur™.

### 2.6. Measurement of Antioxidant Parameters

The treatments for the different cell groups were the same as described for the ROS analysis. For the measurements, the cells were harvested, disrupted ultrasonically in ice, and centrifuged at 2500 ×g for 10 min at 4°C. The supernatants were collected and stored -20°C for subsequent analysis. The activities of catalase (CAT), peroxidase (POD), superoxide dismutase (SOD), and glutathione peroxidase (GSH-Px) were measured using commercial kits following the manufacturer's protocol (Nanjing Jiancheng Bioengineering Institute, Nanjing, China), and the data were normalized to the protein concentration as determined by a BCA protein assay kit.

### 2.7. Real-Time Quantitative RT-PCR

BRL-3A cells were grown in 6-well plates (1 × 10^6^ cells per well) and treated with 0, 1, 10, or 100 *μ*M DHEA for 24 h, then they were exposed to 150 *μ*M H_2_O_2_ for another 2 h. After incubation, the cells were harvested and total RNA was extracted using the TRIZOL reagent kit (Invitrogen, USA) according to the manufacturer's protocols. Total RNA (2 *μ*g) were reverse transcribed into cDNA using the SuperScript II kit (Promega, USA) according to the manufacturer's recommendation. An aliquot of a complementary DNA sample was mixed with 20 *μ*L SYBR Green PCR Master Mix (Roche, Switzerland) in the presence of 10 pmol of each forward and reverse primers for *β-actin* (used as an internal control), *Bcl-2*, and *Bax* (Table 1). All samples were analyzed in duplicate using the iQ5 Sequence Detection System (Bio-Rad, California, USA) and programmed to conduct one cycle (95°C for 3 min) and 40 cycles (95°C for 20 s, 60°C for 30 s, and 72°C for 30 s). The 2^-ΔΔCT^ method was used to calculate the fold change in mRNA levels. The primers used were designed by Primer Premier 5 (Premier Biosoft International, Palo Alto, USA) and synthesized by Invitrogen Biological Co. (Shanghai, China).

### 2.8. Western Blotting

BRL-3A cells were grown in 6-well plates (1 × 10^6^ cells/well) and treated with 50 *μ*M phosphatidylinositol 3-kinase (PI3K) inhibitor (LY294002) or vehicle for 1 h before being exposed to 0, 1, 10, or 100 *μ*M DHEA for 24 h, and then they were treated with 150 *μ*M H_2_O_2_ for another 2 h. The western blotting method was based on our recent description in [23]. Briefly, after incubation, the cells were scraped and the protein concentration was measured using BCA protein determination kits (Beyotime Institute of Biotechnology, Shanghai, China). The extracted protein was separated on 10% sodium dodecyl sulfate-polyacrylamide gel electrophoresis (SDS-PAGE) and transferred onto PVDF membranes (Millipore, Bedford, MA, USA). The membranes were blocked for 3 h with 5% BSA in TBST and then incubated overnight at 4°C with the following rabbit polyclonal antibodies (dilution): PI3K, phosphorylated- (p-) Akt (1 : 1000), Akt (1 : 1000), p-ERK1/2 (1 : 1000), ERK1/2 (1 : 1000), p-JNK (1 : 1000), JNK (1 : 1000), p38 (1 : 500), p-p38 (1 : 500), caspase-3 (1 : 500), and Bax (1 : 5000). After washing with TBST, goat anti-rabbit IgG with horseradish peroxidase conjugated (1 : 10000) in washing solution was added and incubated for 2 h at room temperature. Immunoreactivity proteins were detected by SuperSignal chemiluminescence, and the protein bands were digitally imaged for densitometric quantification using an ECL SuperSignal™ West Pico substrate (Pierce, Rockford, USA). Tubulin *β* monoclonal antibody (1 : 10000) was used as the loading control, and all protein expression levels were normalized to tubulin *β*.

### 2.9. Data Analysis and Statistics

Data were expressed as means ± standard error (SE). Differences were analyzed using one-way analysis of variance (ANOVA) followed by post hoc tests. Differences were considered significant at *P* < 0.05. All statistical analyses were performed with SPSS 20.0 for Windows (StatSoft Inc., Tulsa, USA).

## 3. Results

### 3.1. Biotransformation of DHEA in BRL-3A Cells

**Figure 1:**
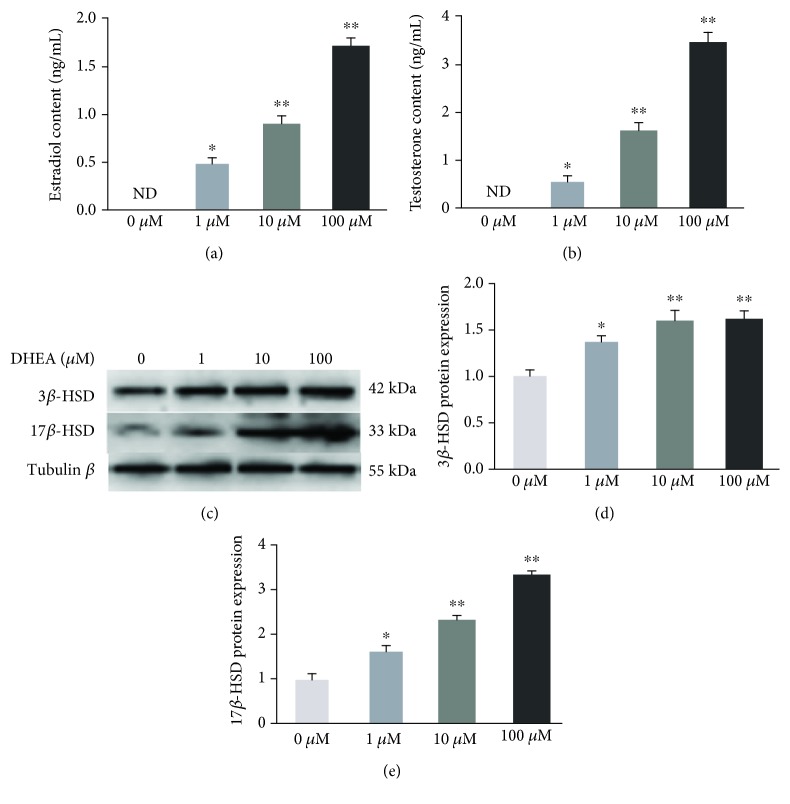
Biotransformation of DHEA in BRL-3A cells. (a) Estradiol content. (b) Testosterone content. (c) Immunoblot of 3*β*-HSD and 17*β*-HSD in BRL-3A cells treated with 0-100 *μ*M DHEA. (d) 3*β*-HSD protein expression. (e) 17*β*-HSD protein expression. Data are presented as the means ± SE. ^∗^*P* < 0.05 and ^∗∗^*P* < 0.01, compared to the vehicle-treated group.

The testosterone and estradiol content were not detected in the vehicle-treated group, while the content of both hormones was significantly higher in the DHEA-treated BRL-3A cells than in the vehicle-treated cells, and it increased as the dose of DHEA increased from 1 to 100 *μ*M (*P* < 0.05) (Figures 1(a) and 1(b)). In keeping with these findings, DHEA treatment resulted in a significant increase in the protein expression of 3*β*-HSD and 17*β*-HSD in a dose-dependent way (*P* < 0.05) (Figures 1(c)–1(e)). These findings are in keeping with the known biotransformation pathways through which DHEA is converted to sex hormones.

### 3.2. DHEA Increased Antioxidant Enzyme Activity in H_2_O_2_-Induced BRL-3A Cells

**Figure 2:**
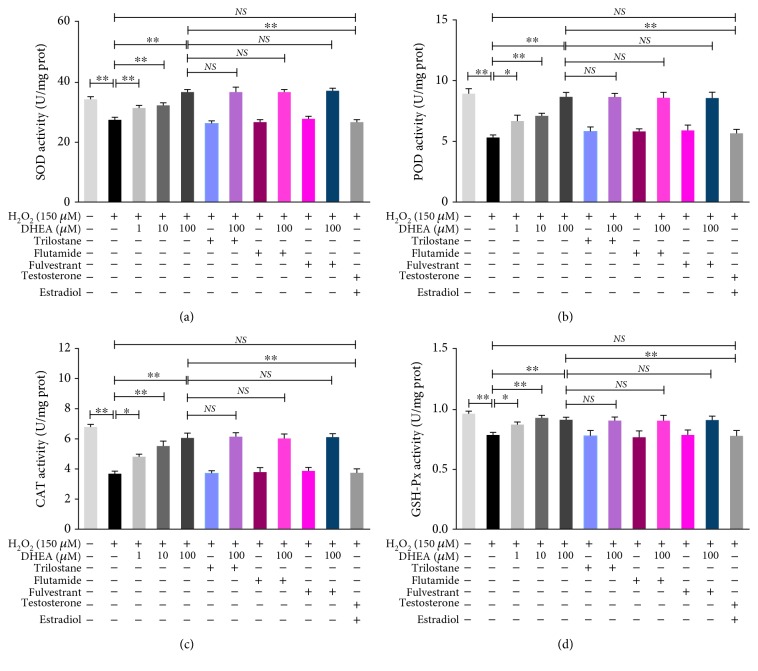
Effects of DHEA on antioxidant enzyme activities in H_2_O_2_-treated BRL-3A cells. (a) Superoxidase dismutase (SOD) activity. (b) Peroxidase (POD) activity. (c) Catalase (CAT) activity. (d) Glutathione peroxidase (GSH-Px) activity. Data are presented as the means ± SE. ^∗^*P* < 0.05 and ^∗∗^*P* < 0.01. NS: no significant difference between the indicated groups.

As shown in Figure 2, the SOD, POD, CAT, and GSH-Px activities were significantly lower in the H_2_O_2_-treated BRL-3A cells than in the vehicle-treated cells (*P* < 0.01). Pretreatment with 1–100 *μ*M DHEA before H_2_O_2_ treatment caused the SOD, POD, CAT, and GSH-Px activities to be significantly higher than those in the cells that were treated only with H_2_O_2_ (*P* < 0.05). These effects of DHEA improved as its dose was increased.

The effects of 100 *μ*M DHEA on the activity of the antioxidant enzymes were not altered when the cells were pretreated with Trilostane (the 3*β*-HSD inhibitor), Flutamide (the AR antagonist), and Fulvestrant (the ER antagonist) (Figure 2). Furthermore, in H_2_O_2_-induced BRL-3A cells that were pretreated with 12.0 nM testosterone and 6.3 nM estradiol, the antioxidant enzyme activities were significantly lower than those in the H_2_O_2_-treated cells that were pretreated with 100 *μ*M DHEA (*P* < 0.01), but there was no significant difference in enzyme activity between H_2_O_2_-treated BRL-3A cells pretreated with 12.0 nM testosterone and 6.2 nM estradiol and the cells treated only with H_2_O_2_ (*P* > 0.05) (Figure 2). The doses of testosterone and estradiol used in this experiment were approximately equal to the estradiol and testosterone concentrations detected in BRL-3A cells that were treated with 100 *μ*M DHEA for 24 h.

### 3.3. DHEA Inhibited H_2_O_2_-Induced ROS Generation in BRL-3A Cells

**Figure 3:**
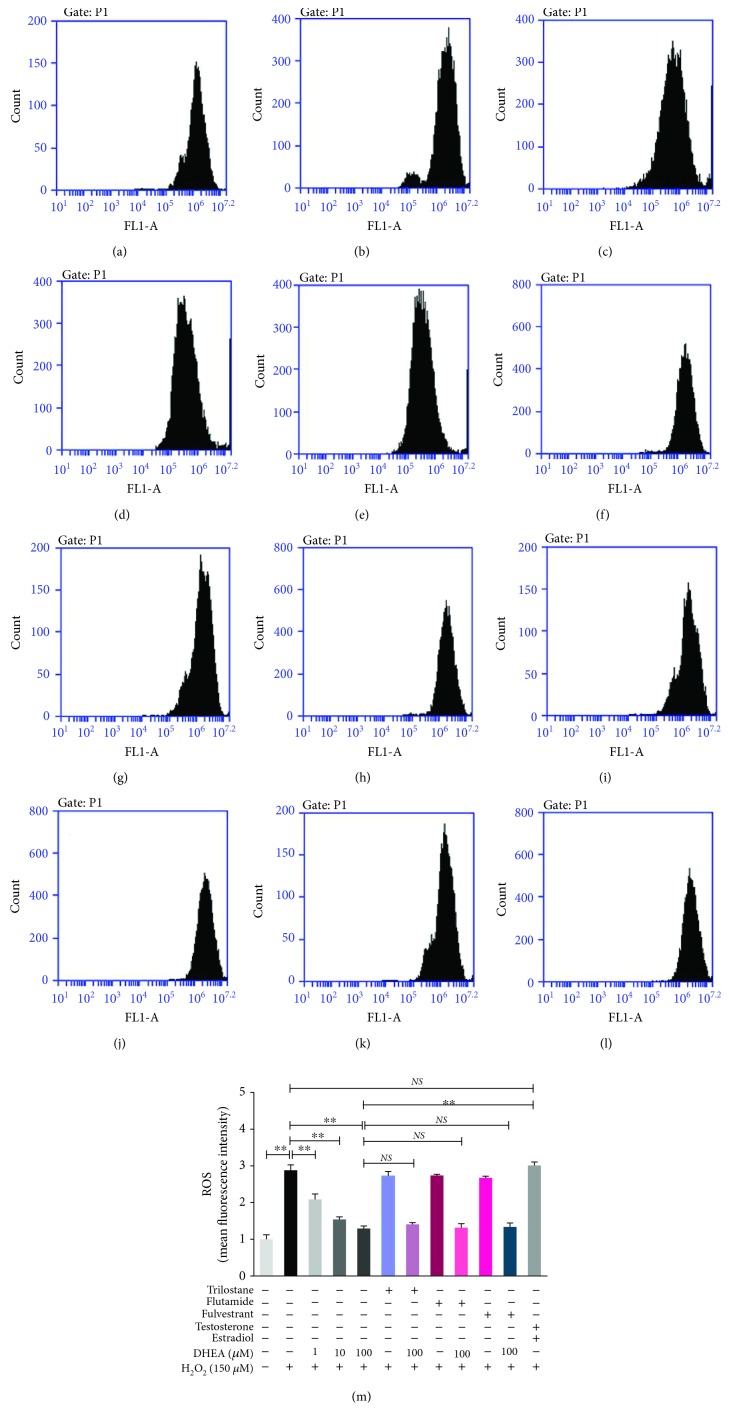
Effects of DHEA on the ROS level in H_2_O_2_-treated BRL-3A cells. (a-l) The flow cytometric histograms of different groups are represented as follows: (a) vehicle-treated group; (b) H_2_O_2_-treated group; (c) 1 *μ*M DHEA and H_2_O_2_-treated group; (d) 10 *μ*M DHEA and H_2_O_2_-treated group; (e) 100 *μ*M DHEA and H_2_O_2_-treated group; (f) Trilostane and H_2_O_2_-treated group; (g) Trilostane, 100 *μ*M DHEA, and H_2_O_2_-treated group; (h) Flutamide and H_2_O_2_-treated group; (i) Flutamide, 100 *μ*M DHEA, and H_2_O_2_-treated group; (j) Fulvestrant and H_2_O_2_-treated group; (k) Fulvestrant, 100 *μ*M DHEA, and H_2_O_2_-treated group; (l) testosterone, estradiol, and H_2_O_2_-treated group. (m) Fluorescence intensities of the different groups. Data are presented as the means ± SE. ^∗∗^*P* < 0.01. NS: no significant difference between the indicated groups.

The ROS content was significantly higher in H_2_O_2_-treated BRL-3A cells than in the vehicle-treated cells (*P* < 0.01). Pretreatment of the H_2_O_2_-treated cells with 1-100 *μ*M DHEA resulted in a significant decrease in the ROS content in a dose-dependent pattern (*P* < 0.01). Pretreatment with Trilostane, Flutamide, or Fulvestrant did not alter these effects of 100 *μ*M DHEA (*P* > 0.05). However, the ROS content in H_2_O_2_-treated BRL-3A cells pretreated with 12.0 nM testosterone and 6.2 nM estradiol was significantly higher than that in the H_2_O_2_-treated cells pretreated with 100 *μ*M DHEA (*P* < 0.01), but it was not significantly different from the ROS content of the cells that were treated only with H_2_O_2_ (*P* > 0.05) (Figure 3).

### 3.4. DHEA Reduced H_2_O_2_-Induced Apoptosis in BRL-3A Cells

**Figure 4:**
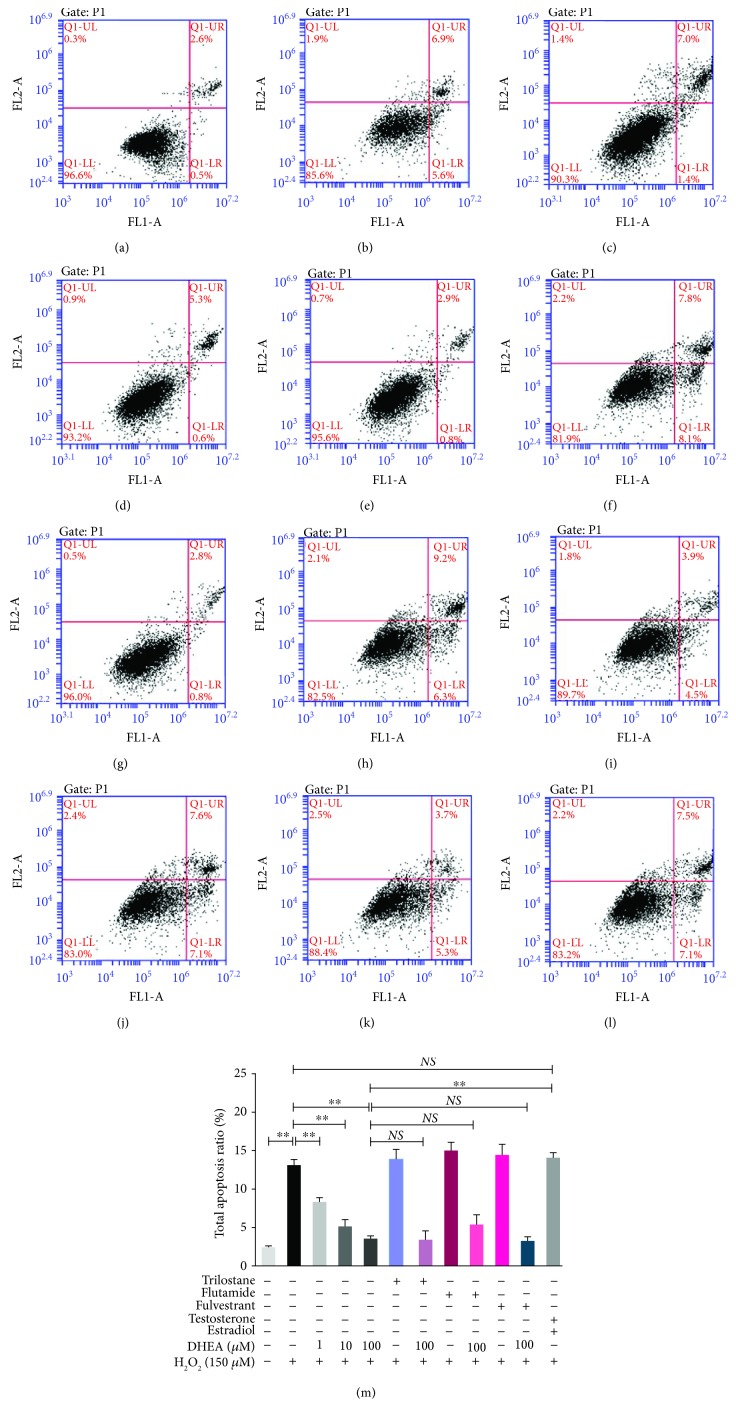
Effect of DHEA on the apoptosis rate of H_2_O_2_-treated BRL-3A cells. (a-l) The flow cytometric histograms of different groups are represented as follows: (a) vehicle-treated group; (b) H_2_O_2_-treated group; (c) 1 *μ*M DHEA and H_2_O_2_-treated group; (d) 10 *μ*M DHEA and H_2_O_2_-treated group; (e): 100 *μ*M DHEA and H_2_O_2_-treated group; (f) Trilostane and H_2_O_2_-treated group; (g) Trilostane, 100 *μ*M DHEA, and H_2_O_2_-treated group; (h) Flutamide and H_2_O_2_-treated group; (i) Flutamide, 100 *μ*M DHEA, and H_2_O_2_-treated group; (j) Fulvestrant and H_2_O_2_-treated group; (k) Fulvestrant, 100 *μ*M DHEA, and H_2_O_2_-treated group; (l) testosterone, estradiol, and H_2_O_2_-treated group. (m) Fluorescence intensities of the different groups. Data are presented as the means ± SE. ^∗∗^*P* < 0.01. NS: no significant difference between the indicated groups.

The apoptosis rate was markedly higher in the H_2_O_2_-treated BRL-3A cells than in the vehicle-treated cells (*P* < 0.01), while it was significantly lower in the H_2_O_2_-treated cells that were pretreated with 1-100 *μ*M DHEA, which showed a dose-dependent effect (*P* < 0.01) (Figure 4). Pretreatment with Trilostane, Flutamide, or Fulvestrant did not alter these anti-apoptotic effects of 100 *μ*M DHEA (*P* > 0.05) (Figure 4). Pretreatment with 12.0 nM testosterone and 6.2 nM estradiol did not change the apoptosis rate of the H_2_O_2_-treated cells (*P* > 0.05). In fact, the apoptosis rate was significantly higher in H_2_O_2_-treated cells pretreated with 12.0 nM testosterone and 6.2 nM estradiol than in H_2_O_2_-treated cells pretreated with 100 *μ*M DHEA (*P* < 0.01) (Figure 4).

### 3.5. DHEA Regulated Apoptosis-Related Factor mRNA Expression in H_2_O_2_-Induced BRL-3A Cells

**Figure 5:**
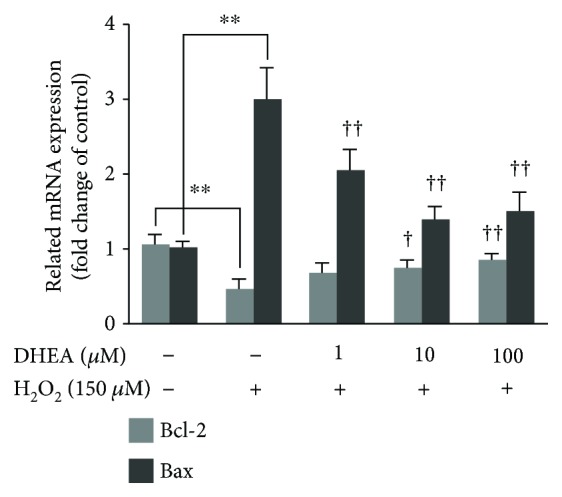
Effect of DHEA on *Bax* and *Bcl-2* mRNA expression in H_2_O_2_-treated BRL-3A cells. Data are presented as the means ± SE. ^∗∗^*P* < 0.01 compared to the vehicle-treated group; ^†^*P* < 0.05 and ^††^*P* < 0.01 compared to the group treated only with H_2_O_2_.

H_2_O_2_-treated BRL-3A cells showed significantly higher *Bax* mRNA expression and significantly lower *Bcl-2* mRNA expression than the vehicle-treated BRL-3A cells (*P* < 0.01). Pretreatment with DHEA at doses of 1-100 *μ*M reversed these effects of H_2_O_2_ on the *Bax* mRNA level (*P* < 0.01), while pretreatment with DHEA at doses of 10-100 *μ*M reversed the inhibitory effect of H_2_O_2_ on *Bcl-2* mRNA expression (*P* < 0.05) (Figure 5).

### 3.6. DHEA Regulated MAPK, PI3K, and p-AKT Protein Level in H_2_O_2_-Induced BRL-3A Cells

**Figure 6:**
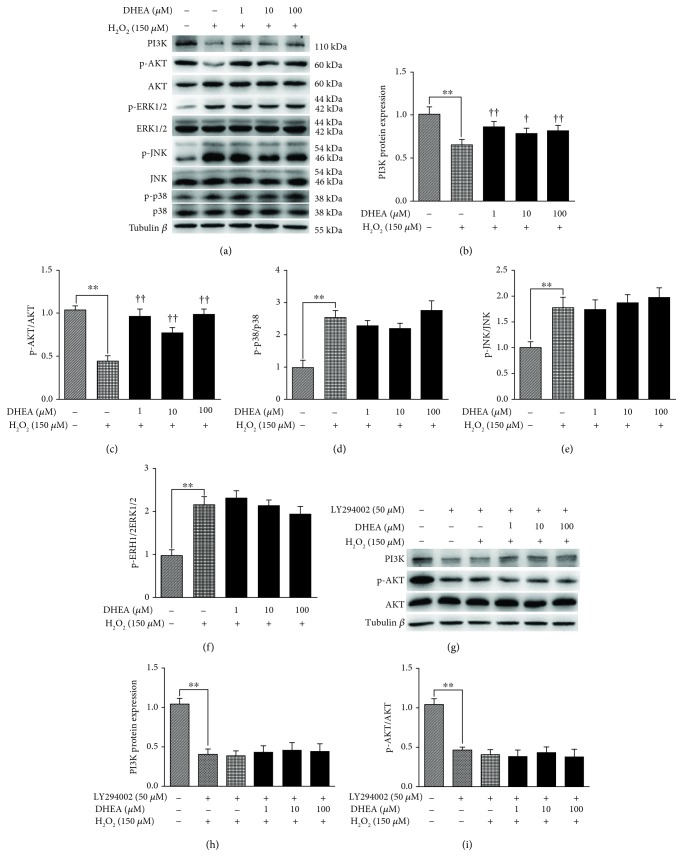
Effects of DHEA on PI3K, p-Akt, and MAPK protein expression in H_2_O_2_-treated BRL-3A cells. (a) Immunoblot of PI3K, p-Akt, and MAPKs in BRL-3A cells pretreated with 1-100 *μ*M DHEA (24 h) and exposed to 150 *μ*M H_2_O_2_ (2 h). (b) PI3K protein expression. (c) p-Akt protein expression. (d) p-p38 protein expression. (e) p-JNK protein expression. (f) p-ERK1/2 protein expression. (g) Immunoblot of PI3K and p-Akt in BRL-3A cells pretreated with 50 *μ*M of the PI3K inhibitor LY294002 or vehicle for 1 h before exposure to 0, 1, 10, or 100 *μ*M DHEA (24 h) and then 150 *μ*M H_2_O_2_ (2 h). (h) PI3K protein expression. (i) p-Akt protein expression. Data are presented as the means ± SE. ^∗∗^*P* < 0.01 compared to the vehicle-treated group; ^†^*P* < 0.05 and ^††^*P* < 0.01compared to the group treated only with H_2_O_2_.

PI3K and p-AKT protein expression was significantly lower in the H_2_O_2_-treated BRL-3A cells than in the vehicle-treated cells (*P* < 0.01) (Figures 6(a)–6(c)), while p-ERK1/2, p-JNK, and p-p38 protein expression was significantly higher (*P* < 0.01) (Figures 6(a) and 6(d)–6(f)). Pretreatment with 1-100 *μ*M DHEA reversed the effects of H_2_O_2_ on PI3K and p-AKT protein expression (*P* < 0.05) (Figures 6(a)–6(c)), but it did not alter the effects of H_2_O_2_ on p-ERK1/2, p-JNK, and p-p38 protein expression (*P* > 0.05) (Figures 6(a) and 6(d)-6(f)). However, these effects of DHEA on PI3K and p-AKT expression were significantly inhibited upon pretreatment of H_2_O_2_-treated cells with LY294002 (*P* < 0.01) (Figures 6(g)–6(i)).

### 3.7. DHEA Downregulated Caspase-3 and Bax Protein Levels in H_2_O_2_-Induced BRL-3A Cells

**Figure 7:**
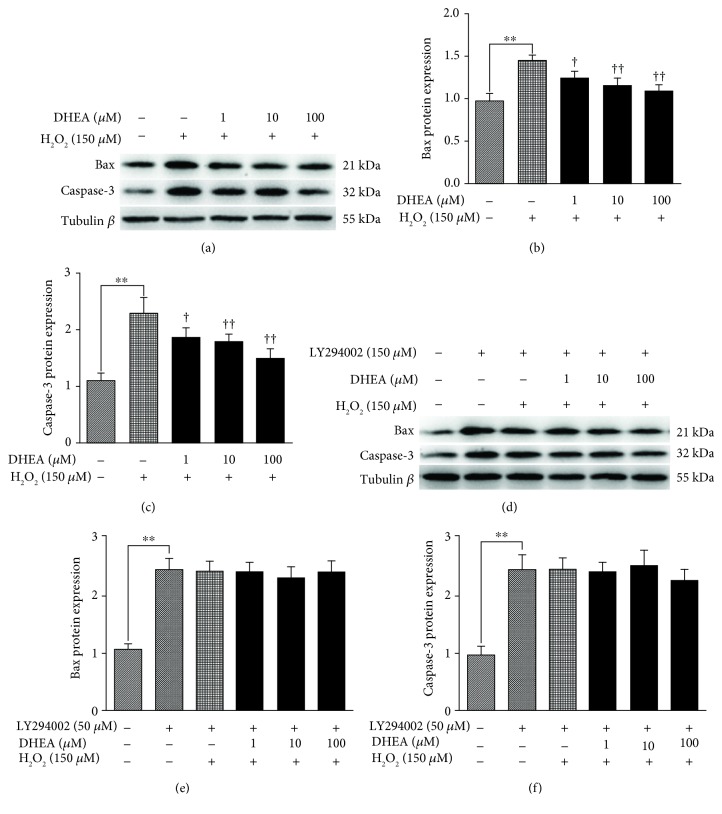
Effects of DHEA on Bax and caspase-3 protein expression in H_2_O_2_-treated BRL-3A cells. (a) Immunoblot of Bax and caspase-3 in BRL-3A cells pretreated with 1-100 *μ*M DHEA (24 h) and then exposed to 150 *μ*M H_2_O_2_ (2 h). (b) Bax protein expression. (c) Caspase-3 protein expression. (d) Immunoblot of Bax and caspase-3 in BRL-3A cells pretreated with 50 *μ*M of the phosphatidylinositol 3-kinase (PI3K) inhibitor LY294002 or vehicle for 1 h before exposure to 0, 1, 10, or 100 *μ*M DHEA (24 h) and then 150 *μ*M H_2_O_2_ (2 h). (e) Bax protein expression. (f) Caspase-3 protein expression. Data are presented as the means ± SE. ^∗∗^*P* < 0.01 compared to the vehicle-treated group; ^†^*P* < 0.05 and ^††^*P* < 0.01 compared to the group treated only with H_2_O_2_.

The protein expression of Bax and caspase-3 was significantly higher in the H_2_O_2_-treated BRL-3A cells than in the vehicle-treated cells (*P* < 0.01) (Figures 7(a)–7(c)). Pretreatment with 1-100 *μ*M DHEA led to a significant decrease in Bax and caspase-3 protein expression in the H_2_O_2_-treated cells (*P* < 0.05) (Figures 7(a)–7(c)). However, these effects of DHEA were inhibited in H_2_O_2_-treated BRL-3A cells that were pretreated with LY294002 (*P* < 0.05) (Figures 7(d)–7(f)).

## 4. Discussion

In the present study, our *in vitro* experiments on rat liver cells show that DHEA exerts antioxidative and antiapoptotic effects through pathways that are independent of steroid hormones and their receptors; instead, the PI3K/p-AKT pathways seem to be closely involved with these protective effects of DHEA on the liver cells.

BRL-3A rat liver cells were used as an *in vitro* model for studying the effects of H_2_O_2_, as this cell type is susceptible to oxidative damage. Furthermore, H_2_O_2_ is well known as an ROS that easily penetrates cells and reacts with metal ions to produce highly reactive hydroxyl radicals that can cause severe cellular injuries. Here, exposure to 150 *μ*M of H_2_O_2_ led to the loss of antioxidant activity and generation of ROS in the BRL-3A cells. Similarly, H_2_O_2_ has been shown to induce oxidative damage via its inhibitory effects on antioxidant enzyme activity [27, 28]. Furthermore, in the present study, treatment with DHEA reversed these effects of H_2_O_2_. This is also in keeping with the findings of previous studies in which DHEA was reported to inhibit *in vitro* glucose-induced ROS generation [29] and *in vivo* ROS generation, as well as to increase the *in vivo* protein expression of NADPH oxidase [30]. In particular, DHEA was found to exert antioxidant effects by inducing an increase in catalase expression, activating the thioredoxin system, and suppressing superoxide anion production [14, 31]. Also in this study, DHEA was found to promote the activity of the antioxidant enzymes such as SOD, CAT, POD, and GSH-Px in the H_2_O_2_-treated cells. Thus, all these findings demonstrate that DHEA protects the cell against oxidative damage by promoting the activity of antioxidant enzymes and inhibiting ROS production.

Another target of DHEA is cellular apoptotic pathways, as we found that BRL-3A cells that were pretreated with DHEA were protected against the apoptosis-promoting effects of H_2_O_2_. Among the proteins involved in apoptosis, Bax and Bcl-2, which are members of the Bcl family of proteins, play an important role as regulators of the initial stages of apoptosis [32]. In this study, DHEA inhibited the H_2_O_2_-induced downregulation of Bcl-2 mRNA expression and upregulation of Bax mRNA expression. Caspases are also important players in the apoptosis pathways, and activated caspase-3, in particular, plays a major proapoptotic role [33]. Our results showed that pretreatment with DHEA led to a significant decrease in the protein expression of Bax and caspase-3 in BRL-3A cells that were treated with H_2_O_2_. Therefore, DHEA might protect liver cells from the effects of H_2_O_2_ through its regulatory effects on downstream apoptosis-related proteins of the Bcl-2 and caspase family.

As DHEA has been reported to exert its physiological effects via bioconversion into sex hormones and activation of estrogen/androgen receptors, we also investigated whether DHEA exerted its antioxidative effects via such hormonal mechanisms. First, direct measurement of the testosterone and estradiol contents showed that they had increased in the BRL-3A cells after DHEA treatment. Consistent with these changes in the active hormone content, a significant increase was observed in the protein expression of 3*β*-HSD and 17*β*-HSD after DHEA treatment. In agreement with our findings, DHEA has been shown to promote the testosterone and estradiol levels in both *in vivo* and *in vitro* settings [34, 35]. These findings can be explained through the process of bioconversion wherein 3*β*-HSD catalyzes the rapid conversion of DHEA into androstenedione in peripheral organs such as the brain, liver, kidney, and gonads, after which androstenedione is converted into testosterone and estradiol by 17*β*-HSD and aromatase, respectively [36]. However, although there was evidence of the bioconversion of DHEA, we found that pretreating H_2_O_2_-treated BRL-3A cells with Trilostane (a 3*β*-HSD inhibitor), Flutamide (an AR antagonist), and Fulvestrant (an ER antagonist) did not alter the effects of DHEA on the ROS content, antioxidant enzyme activity, or apoptosis rate. Furthermore, pretreatment with testosterone and estradiol did not have the same effects as pretreatment with DHEA on the ROS content, antioxidant activity, or apoptosis rate of H_2_O_2_-treated BRL-3A cells. From these findings, it can be speculated that although a small amount of DHEA was converted into active steroid hormones in BRL-3A cells, the antioxidant and antiapoptotic effects of DHEA were independent of steroid hormonal mechanisms.

PI3K/Akt and mitogen-activated protein kinases (MAPKs) such as ERK1/2, JNK, and p38 are known to participate in oxidative stress-induced cell apoptosis [37]. Specifically, MAPKs were found to play a proapoptotic role and PI3K/Akt was found to play a prosurvival role in cells exposed to oxidative stress [38]. Based on these findings, we analyzed the expression of these proteins in H_2_O_2_-treated BRL-3A cells that were protected by DHEA pretreatment. There was a significant increase in the protein expression of p-ERK1/2, p-JNK, and p-p38 after H_2_O_2_ treatment, but pretreatment with DHEA did not alter these effects of H_2_O_2_. Thus, the antioxidant mechanisms of DHEA may not involve the activation of MAPK signaling pathways. However, previous studies have shown that DHEA induced a decrease in the level of phosphorylated JNK and p38 in H_2_O_2_-treated muscle cells and thereby decreased their apoptosis rate [39]. The differences in the findings might be related to the use of different cell types in the studies.

In contrast to the findings on MAPK signaling, in the current study, DHEA pretreatment resulted in a significant decrease in the protein expression of PI3K and p-Akt in the H_2_O_2_-treated cells. In agreement with our findings, it has been reported that DHEA protects human neuroblastoma SH-SY5Y cells from apoptosis induced by serum deprivation via the PI3K/Akt pathway [40]. Furthermore, in our study, pretreatment with DHEA also repressed the increase in the protein expression of Bax and caspase-3 that was induced by H_2_O_2_. These effects of DHEA on the expression of PI3K, p-Akt, Bax, and caspase-3 were eliminated when the cells were pretreated with the PI3K inhibitor LY294002. This confirms the involvement of the PI3K/Akt pathways in the DHEA-mediated protection of BRL-3A cells against apoptosis induced by H_2_O_2_. Based on all the findings, it can be concluded that DHEA protected BRL-3A cells against H_2_O_2_-induced oxidative damage by activating PI3K/Akt signaling pathways, rather than by activating MAPK signaling pathways.

**Figure 8:**
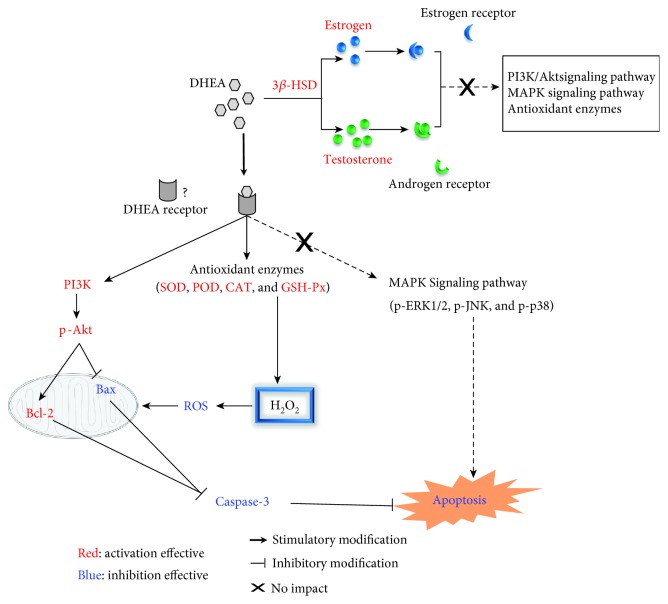
Schematic diagram of the potential mechanisms through which DHEA protects H_2_O_2_-treated BRL-3A cells from oxidative damage and apoptosis. There are at least three parallel regulation mechanisms that contribute to the protective effects of DHEA on H_2_O_2_-treated BRL-3A cells: (1) increasing cellular antioxidative enzyme activities, thus decreasing the levels of intracellular reactive oxygen species and reducing oxidative damage; (2) reducing caspase-3 protein levels through the activation of PI3K/Akt signaling pathways, rather than MAPK signaling pathways; and (3) activating specific receptors rather than androgen and estrogen receptors.

In conclusion, our data demonstrate that DHEA exerted a protective effect on BRL-3A cells that were exposed to H_2_O_2_ by inhibiting the production of ROS, promoting the activity of antioxidant enzymes, and regulating the expression of apoptosis-related proteins. Furthermore, the antiapoptosis mechanisms of DHEA involved the activation of the PI3K/Akt signaling pathways, rather than the MAPK signaling pathways. Importantly, these effects of DHEA are independent of androgen and estrogen receptor pathways (Figure 8). This could mean that DHEA directly interacts with specific receptors to exert these effects, but this needs to be explored through future investigations into its mechanisms. This information not only increases our understanding of the molecular mechanisms of DHEA, but it also highlights the potential applications of DHEA in the treatment of diseases, especially liver diseases, caused by oxidative stress.

## Figures and Tables

**Table 1:** Primer sequences of the target genes and *β*-actin (internal control) used for RT-PCR.

Gene	GenBank accession number	Primer sequences (5′-3′)	Orientation	Product size (bp)
*β-Actin*	NM 031144	CCCTGTGCTGCTCACCGA ACAGTGTGGGTGACCCCGTC	Forward Reverse	186

*Bax*	NM 007527	GCAGGGAGGATGGCTGGGGAGA TCCAGACAAGCAGCCGCTCACG	Forward Reverse	352

*Bcl-2*	NM 016993	CGACTTTGCAGAGATGTCCA CATCCACAGAGCGATGTTGT	Forward Reverse	202

## Data Availability

The data used to support the findings of this study are available from the corresponding author upon request.
